# Derivative-Free Power Flow Solution for Bipolar DC Networks with Multiple Constant Power Terminals

**DOI:** 10.3390/s22082914

**Published:** 2022-04-11

**Authors:** Ángeles Medina-Quesada, Oscar Danilo Montoya, Jesus C. Hernández

**Affiliations:** 1 Department of Electrical Engineering, University of Jaén, Campus Lagunillas s/n, Edificio A3, 23071 Jaén, Spain; jcasa@ujaen.es; 2 Grupo de Compatibilidad e Interferencia Electromagnética, Facultad de Ingeniería, Universidad Distrital Francisco José de Caldas, Bogotá 110231, Colombia; 3 Laboratorio Inteligente de Energía, Facultad de Ingeniería, Universidad Tecnológica de Bolívar, Cartagena 131001, Colombia

**Keywords:** power flow solution, bipolar DC networks, monopolar and bipolar constant power loads, triangular-based formulation, convergence evaluation

## Abstract

This paper analyzes the power flow solution in bipolar direct current networks with radial structures considering multiple monopolar and bipolar constant power loads. The electrical configuration of the bipolar DC grid considers that the reference pole is non-grounded along the feeder, which produces important neutral currents and voltage imbalances along the DC grid. The power flow problem is formulated through the triangular-based representation of the grid topology, which generates a recursive formulation that allows determining the voltage values in the demand nodes through an iterative procedure. The linear convergence of the triangular-based power flow method is tested through multiple load variations with respect to the nominal grid operative condition. Numerical results in the 21- and the 85-bus grids reveal the relevant variations in the voltage profiles and total grid power losses when the neutral cable is solidly grounded or not.

## 1. Introduction

Recently, the direct current (DC) distribution network has gained attention in the research and industrial communities since they do not require generation synchronization, have better voltage profiles, present lower power and energy losses, and in these grids, reactive power and frequency concepts are nonexistent [1,2]. Additionally, the accelerated advances in power electronic converters make these grids attractive for integrating multiple distributed energy resources such as photovoltaic generation, battery energy storage systems, and fuel cells, since these operate directly with DC technology, reducing the number of converters required in comparison with classical alternating current (AC) networks [3,4,5].

Electrical distributions with DC technologies can be constructed with two possible configurations: monopolar and bipolar [6,7]. The former is the most typical structure, where a positive and a neutral pole are used to provide constant voltage to multiple loads connected in the feeder [8]. The latter, i.e., bipolar, is composed of three cables that work as positive, neutral, and negative poles, respectively [9,10]. The main characteristic of the bipolar connection is that it allows the transfer of double the power to the loads in comparison to the monopolar connection solely by including a new cable. Further, under a perfectly balanced load case, the neutral cable can be eliminated, which reduces about 33% of the total investment costs in conductors [11]. An additional advantage of the bipolar connection is that for certain special loads, it is possible to duplicate the voltage profile provided to the load by interconnection between the positive and negative poles [12].

To examine the DC distribution under steady-state conditions, even if this works with monopolar or bipolar configurations with multiple constant power loads, it is mandatory to implement the power flow methodologies owing to the nonlinearities introduced by the constant power terminals [13,14]. In the case of monopolar configurations in the current literature, multiple approaches based on derivative-free and derivative-based methods are found, such as backward/forward, successive approximations, triangular-based, Newton–Raphson, and Taylor-based power flow methods [15]. For bipolar DC grids, few works have recently been developed for power flow and optimal power flow analyses in these networks, some of which are discussed below. Authors of [9] proposed a mixed-integer linear multi-objective optimization model for phase-swapping in the bipolar DC grids; however, they do not consider the existence of constant power loads, which is an oversimplification of the power flow model. Authors of [16] presented an optimal power flow formulation for bipolar DC networks with multiple constant power loads, including dispersed generators; additionally, they incorporated the effect of the neutral pole and its resistance in their analysis. The objective of this work was to compute the locational marginal prices of the network; however, to obtain these, the authors have linearized the hyperbolic constraints regarding constant power loads, which implies that the power flow problem was again simplified, as in the previous work. Authors of [17] illustrated an admittance nodal formulation to solve the power flow problem in bipolar DC grids with constant power terminals. A numerical evaluation into a three-bus system is made using the PSCAD/EMTDC software; however, they do not present any analysis of the model complexity or convergence test under load variations, only altering the constant power terminals for controlled current sources and leaving the complications of the solution to a power system analyzer tool. In reference [18], the authors studied the optimal power flow problem in DC bipolar distribution networks considering high load unbalanced. The authors proposed a linearized optimal power flow model to determine the effect of the grid congestion on the local marginal prices per node. Numerical results in two example test feeders show the effectiveness of the proposed approach; however, the authors do not make any comparison with other alternative optimal power approaches. Authors of [10] presented an optimal power flow formulation with multiple load unbalances by applying the current injection method. Numerical results showed the effectiveness of the proposed formulation by using a sensitivity analysis based on the Jacobian matrix to determine the costs of the total grid unbalance. Their numerical achievements were verified with the help of the PSCAD/EMTDC software.

Owing to the existing gap in the current literature regarding numerical methodologies in solving the power flow problem in bipolar DC grids with constant power terminals, this research proposes the extension of the triangular-based power flow method expounded initially in [16] for bipolar configurations, considering that the neutral pole can or cannot be solidly grounded in all the nodes of the network. A recursive power flow formula is derived, and it can be easily applied for systems with multiple monopolar loads and pure bipolar loads even if high imbalances are present. The numerical results in the 21- and the 85-bus systems portray the negative effect of non-grounding the neutral cable, since important voltage values appear in the neutral pole added with important increments in the total power losses. Moreover, to show the linear convergence properties of the proposed power flow method, multiple load scenarios were considered.

This document is organized as follows: Section 2 presents the derivation of the recursive power flow formula based on the upper-triangular matrix representation; Section 3 unveils the iterative procedure to solve the power flow problem in bipolar DC grids; Section 4 reveals the main characteristics of the 21-bus and the 85-bus grid system. Section 5 details the numerical validations and their analyses and discussions. Finally, Section 6 describes the main concluding remarks and some possible future works derived from this research.

## 2. Power Flow Formulation

**Figure 1:**
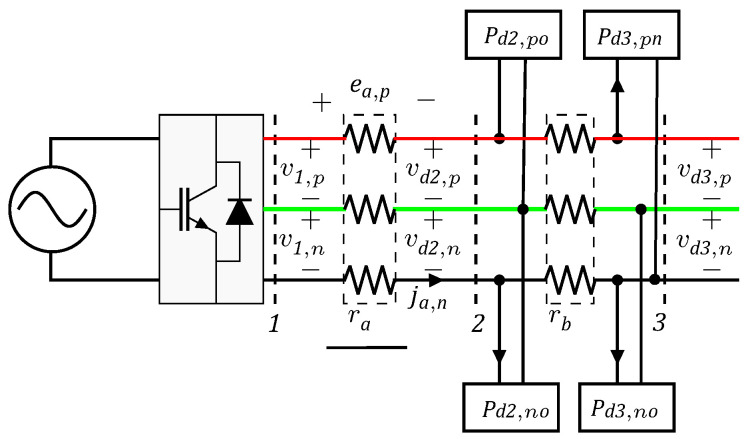
Schematic representation of a bipolar DC grid.

A bipolar DC distribution network with multiple constant power loads can be analyzed through a recursive formulation that allows the determining of the final voltage profile in the positive *p*, neutral *o*, and negative *n* poles from an initial voltage value provided in the first iteration [19]. To illustrate the formulation of the triangular-based bipolar power flow method, let us consider a small bipolar network composed of three nodes, depicted in Figure 1 [12].

Figure 1 shows that the nodes can be connected with multiple constant power terminals. Note that in an arbitrary node *k*, it is possible to have loads connected between the positive and neutral poles, i.e., $p_{dk,po}$, loads between the negative and neutral poles, i.e., $p_{dk,no}$, and also pure bipolar loads named $p_{dk,pn}$. It is worth mentioning that the index *d* is used in referring to demand nodes.

Now, if the first Kirchhoff’s law is applied to each node, where the current through the line *l* is named $j_{l,pon}={\left[j_{l,p},\;j_{l,o},\;j_{l,n}\right]}^{⊤}$ and the demanded current at node *k* is defined as $i_{dk,pon}={\left[i_{dk,p},\;i_{dk,o},\;i_{dk,n}\right]}^{T}$, the following set of linear equations are formed:

(1) $$\begin{array}{c} \left[\begin{array}{c} j_{a,p}\\ j_{a,o}\\ j_{a,n}\\ j_{b,p}\\ j_{b,o}\\ j_{b,n} \end{array}\right]=\left[\begin{array}{cccccc} 1 & 0 & 0 & 1 & 0 & 0\\ 0 & 1 & 0 & 0 & 1 & 0\\ 0 & 0 & 1 & 0 & 0 & 1\\ 0 & 0 & 0 & 1 & 0 & 0\\ 0 & 0 & 0 & 0 & 1 & 0\\ 0 & 0 & 0 & 0 & 0 & 1 \end{array}\right]\left[\begin{array}{c} i_{d2,p}\\ i_{d2,o}\\ i_{d2,n}\\ i_{d3,p}\\ i_{d3,o}\\ i_{d3,n} \end{array}\right], \end{array}$$

which can be easily compacted in a matricial structure with the form defined in Equation (2).

(2) $$\begin{array}{c} J_{l,pon}=T_{ll,pon}I_{d,pon}, \end{array}$$

where $J_{l,pon}\in R^{3l\times 1}$ is the vector that contains all the branch currents, $i_{d,pon}\in R^{3(b-1)\times 1}$ is the vector that comprises all of the demanded currents (*b* being the total number of nodes/buses the network), and $T_{ll,pon}\in R^{3l\times 3l}$ is defined as the triangular matrix that relates branches with demand currents. Note that the triangular-based formulation defined in (2) is only applicable to DC bipolar networks with radial structures and a unique voltage controlled source (slack node).

Now, if the second Kirchhoff’s law is applied to each closed-loop trajectory from the slack node to each demand node, then the following set of linear equations is obtained:

(3) $$\begin{array}{c} \left[\begin{array}{c} v_{d2,p}\\ v_{d2,o}\\ v_{d2,n}\\ v_{d3,p}\\ v_{d3,o}\\ v_{d3,n} \end{array}\right]=\left[\begin{array}{ccc} 1 & 0 & 0\\ 0 & 1 & 0\\ 0 & 0 & 1\\ 1 & 0 & 0\\ 0 & 1 & 0\\ 0 & 0 & 1 \end{array}\right]\left[\begin{array}{c} v_{s,p}\\ v_{s,o}\\ v_{s,n} \end{array}\right]-\left[\begin{array}{cccccc} 1 & 0 & 0 & 0 & 0 & 0\\ 0 & 1 & 0 & 0 & 0 & 0\\ 0 & 0 & 1 & 0 & 0 & 0\\ 1 & 0 & 0 & 1 & 0 & 0\\ 0 & 1 & 0 & 0 & 1 & 0\\ 0 & 0 & 1 & 0 & 0 & 1 \end{array}\right]\left[\begin{array}{c} e_{a,p}\\ e_{a,o}\\ e_{a,n}\\ e_{b,p}\\ e_{b,o}\\ e_{b,n} \end{array}\right], \end{array}$$

which can be compacted in its matricial form as follows:

(4) $$\begin{array}{c} V_{d,pon}=A_{ds}V_{s,pon}-T_{ll,pon}^{⊤}E_{l,pon}, \end{array}$$

where $V_{d,pon}\in R^{3(b-1)\times 1}$ is the vector that contains all the voltage profiles in the positive, neutral, and negatives poles for the demand nodes, $V_{s,pon}\in R^{3s\times 1}$ is the vector with the voltage outputs in the slack source (known voltage values), $E_{l,pon}R^{3l\times 1}$ is the vector that consists of all the voltage drops in the grid branches, and $A_{ds}\in R^{3(b-1)\times 3}$ is a tri-diagonal matrix composed of identity diagonal matrices that relate each demand node with the substation.

To relate branch current and branch voltage drops, Ohm’s law is applied to each branch *l*, which produces the following set of linear equations:

(5) $$\begin{array}{c} \left[\begin{array}{c} e_{a,p}\\ e_{a,o}\\ e_{a,n}\\ e_{b,p}\\ e_{b,o}\\ e_{b,n} \end{array}\right]=\left[\begin{array}{cccccc} r_{a,p} & 0 & 0 & 0 & 0 & 0\\ 0 & r_{a,o} & 0 & 0 & 0 & 0\\ 0 & 0 & r_{a,n} & 0 & 0 & 0\\ 0 & 0 & 0 & r_{b,p} & 0 & 0\\ 0 & 0 & 0 & 0 & r_{b,o} & 0\\ 0 & 0 & 0 & 0 & 0 & r_{b,n} \end{array}\right]\left[\begin{array}{c} j_{a,p}\\ j_{a,o}\\ j_{a,n}\\ j_{b,p}\\ j_{b,o}\\ j_{b,n} \end{array}\right], \end{array}$$

which can be compacted as defined in Equation (6)

(6) $$\begin{array}{c} E_{l,pon}=R_{ll,pon}J_{l,pon}, \end{array}$$

where $R_{ll,pon}\in R^{3l\times 3l}$ is a diagonal matrix that contains all the resistive effects of the grid branches.

Now, if Equation (2) is substituted in Equation (6) and its result is replaced in Equation (4), consequently, the triangular-based power flow formula for bipolar DC networks is obtained as defined in (7).

(7) $$\begin{array}{c} V_{d,pon}=A_{ds}V_{s,pon}-T_{ll,pon}^{⊤}R_{ll,pon}T_{ll,pon}I_{d,pon}, \end{array}$$

For simplicity in the formulation, the resistance-like nodal matrix, $R_{dd,pon}$, as $T_{ll,pon}^{⊤}$$R_{ll,pon}T_{ll,pon}$, is defined to convert Equation (7) into (8).

(8) $$\begin{array}{c} V_{d,pon}=A_{ds}V_{s,pon}-R_{dd,pon}I_{d,pon}. \end{array}$$

Note that Equation (8) is indeed a linear relation between demanded voltages and currents; however, due to the presence of multiple constant power terminals, this equation transformed into a general nonlinear non-convex equation. From Figure 1, it is possible to define the current demanded in an arbitrary node *k* for each pole as follows [17]:  

(9) $$\begin{array}{c} i_{dk,p}=\frac{p_{dk,po}}{v_{dk,p}-v_{dk,o}}+\frac{p_{dk,pn}}{v_{dk,p}-v_{dk,n}}, \end{array}$$

(10) $$\begin{array}{c} i_{dk,o}=\frac{p_{dk,po}}{v_{dk,o}-v_{dk,p}}+\frac{p_{dk,no}}{v_{dk,o}-v_{dk,n}}, \end{array}$$

(11) $$\begin{array}{c} i_{dk,n}=\frac{p_{dk,no}}{v_{dk,n}-v_{dk,o}}+\frac{p_{dk,pn}}{v_{dk,n}-v_{dk,p}}. \end{array}$$

To arrive at a compact formula for the calculation of the demand current at each node, as defined in Equations (9)–(11), let us define the auxiliary variables that measure the voltage difference between poles as follows: $v_{dk,po}=v_{dk,p}-v_{dk,o}$, $v_{dk,pn}=v_{dk,p}-v_{dk,n}$, and $v_{dk,no}=v_{dk,n}-v_{dk,o}$, which can be grouped into a vector as $\Delta v_{dk,pon}={\left[v_{dk,po},\;v_{dk,pn},\;v_{dk,no}\right]}^{⊤}$. With these definitions, the demanded current in the *k* node can be calculated as presented below:

(12) $$\begin{array}{c} I_{dk,pon}=H{\mathrm{diag}}^{-1}\left(\Delta v_{dk,pon}\right)P_{dk,pon}, \end{array}$$

where

$$\begin{array}{c} I_{dk,pon}=\left[\begin{array}{c} i_{dk,p}\\ i_{dk,o}\\ i_{dk,n} \end{array}\right],H=\left[\begin{array}{ccc} 1 & 1 & 0\\ -1 & 0 & -1\\ 0 & -1 & 1 \end{array}\right],P_{dk,pon}=\left[\begin{array}{c} p_{dk,po}\\ p_{dk,pn}\\ p_{dk,no} \end{array}\right]. \end{array}$$

It is worth mentioning that in finding all the demanded voltages with Equation (8), it is necessary to use an iterative procedure since the demanded currents at each node *k* defined in (12) are a function of the demanded voltages, which implies that in order to know these voltages, it is necessary to have known them previously, which is only solvable through a recursive implementation, as will be presented in the next section [19].

## 3. Iterative Solution

To solve the power flow problem in bipolar DC grids with multiple constant power loads, the iterative procedure implemented is presented in Algorithm 1.

In Algorithm 1, the parameter ζ is the convergence error between two consecutive voltages, which is defined as recommended in [19] as $1\times 10^{-10}$. Note that once the power flow problem is solved by implementing the Algorithm 1, the total grid power losses in all the branches of the network can be determined as follows:

(13) $$\begin{array}{c} p_{\mathrm{loss}}=J_{l,pon}^{⊤}R_{ll,pon}J_{l,pon}, \end{array}$$

**Algorithm 1:** Power flow solution using the triangular-based formulation for multiple constant power loads.
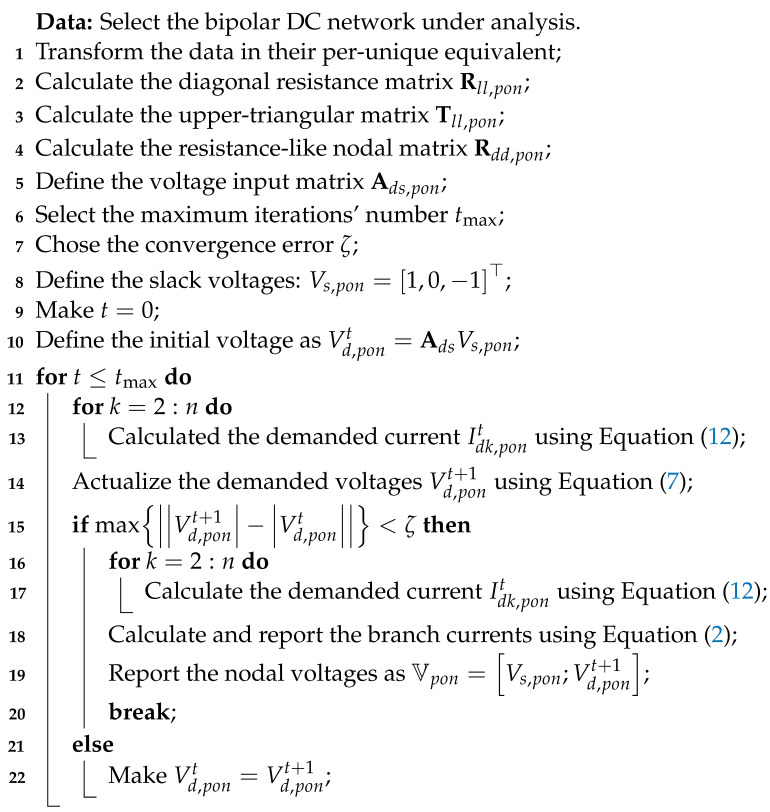



## 4. Test Feeders

To evaluate the efficiency of the proposed triangular-based power flow approach for bipolar DC grids with multiple constant power loads, two test feeders are employed. The characteristic of each one of these test feeders is presented below.

### 4.1. 21-Bus System

**Figure 2:**
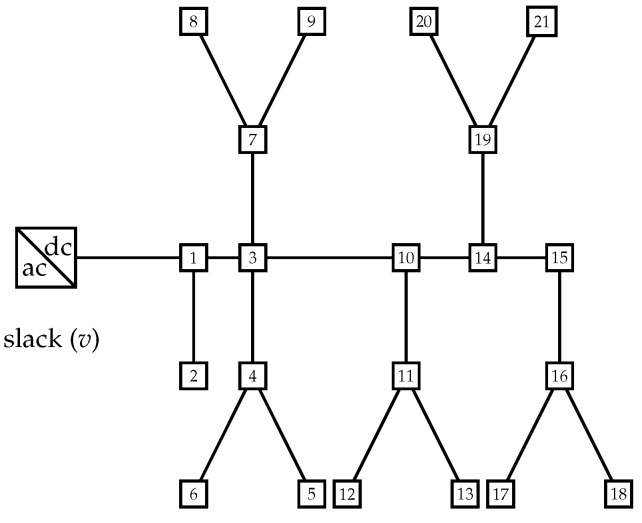
Grid configuration of the 21-bus system.

The 21-bus system was adapted from [19] to include loads connected to the positive and negative poles with respect to the neutral as well as pure bipolar loads. The electrical configuration of this grid is depicted in Figure 2.

For this test feeder, the slack bus works in the positive and negative poles with ±1 kV. All the electrical parameters regarding branches and loads are reported in Table 1. Notably, in this research, we assume that all the poles, including the neutral, were constructed with the same caliber.

### 4.2. 85-Bus System

**Figure 3:**
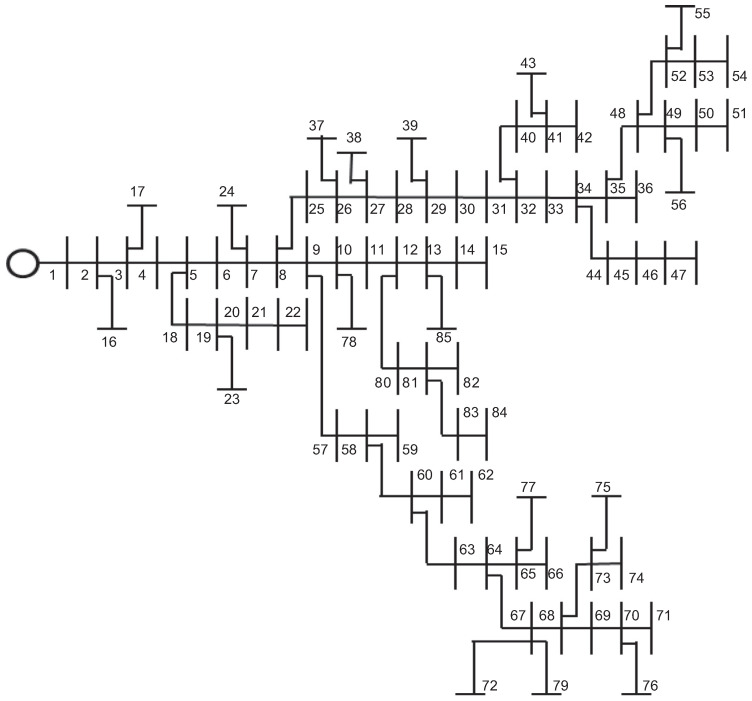
Grid topology of the IEEE 85-bus system for bipolar power flow applications.

The 85-bus system is a DC bipolar adaptation of the IEEE 85-bus system presented in [20] to locate and size fixed-step capacitor banks. This system is operated with 11 kV per pole, and it has radial configuration, i.e., 84 distribution lines. The electrical configuration of this test feeder is depicted in Figure 3.

The complete parametric information for the 85-bus grid with bipolar structure is presented in Table 2.

## 5. Numerical Validations

The numerical implementation of the proposed triangular-based bipolar power flow method was executed in the MATLAB programming environment using the researcher’s own scripts. For this implementation, 2021b was used on a PC with an AMD Ryzen 7 3700 2.3 GHz processor and 16.0 GB RAM, running on a 64-bit version of Microsoft Windows 10 Single language.

### 5.1. Results in the 21-Bus System

To demonstrate the effectiveness of the proposed method on addressing the power flow solution in bipolar DC grids with multiple constant power loads, we consider two simulation cases regarding the neutral pole: (i) this pole is solidly grounded at each one of the nodes of the network; and (ii) this pole is only solidly grounded at the substation bus.

Table 3 exhibits the voltage in the positive and negative poles when both cases are simulated.

The numerical data provided in Table 3 permit noting the following: (i) in the first simulation case when the neutral is solidly grounded, the positive pole presents some nodes with voltage regulations more than 10%, presenting a minimum voltage at node 17 with a magnitude of 890.1027 V, which implies that the grid regulation is about 10.99%. Conversely, for the negative pole, the worst regulation voltage appears at node 18 with a value of 9.14%. The voltage unbalance between the positive and negative poles is caused by the load disequilibrium, since the positive pole has about 554 kW of constant power consumption while the negative pole has only 445 kW of the same. (ii) In the second case, when the neutral pole is not connected to the electrical ground at each node, it is possible to observe that nodes 15 to 18 have voltages higher than 18 V, which can cause the misoperation of some devices in the adjacent areas, especially when sensitive to voltage variations on the reference point. Additionally, the non-grounded neutral pole can worsen the voltage profile in some nodes of the network, as seen for the positive voltage profile at node 17, where the voltage has decreased 1.8433 V with respect to the first simulation case.

The main issue when the neutral pole is not solidly grounded in all the nodes corresponds to the total grid power losses since these are increased with respect to the solidly grounded case. For example, in the 21-bus system for the first simulation case, the total power losses are 91.2701 kW, while for the second, this number increased to 95.4237 kW. These values indicate that there is about 4.1536 kW that is transformed into heat owing to the currents flowing through the neutral cable.

**Figure 4:**
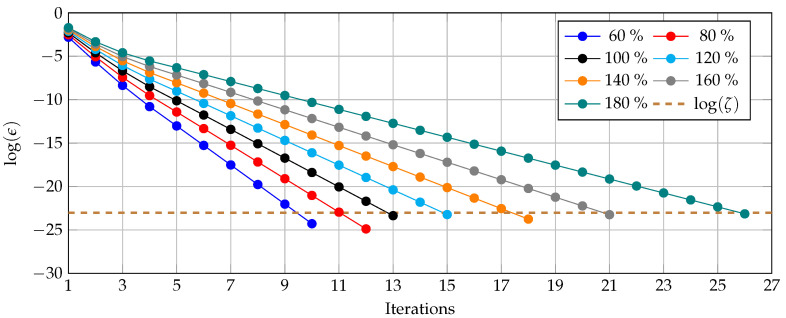
Convergence rate of the studied method for load variations in the 21-bus system.

As evidence of the convergence properties of the studied triangular-based bipolar power flow method, we plot the logarithm value of the convergence error, i.e., $\log\left(\epsilon \right)$ (where $\epsilon =\max\left\{\left|\left|V_{d,pon}^{t+1}\right|-\left|V_{d,pon}^{t}\right|\right|\right\}$), for the worst operative case (i.e., non-grounded simulation scenario) considering that the load varies from 60% to 180% of their nominal values listed in Table 1. The convergence rate is depicted in Figure 4. Note that the 100% corresponds to the benchmark case of the network where the load takes its nominal value.

The numerical performance of the triangular-based method for bipolar DC grids in the 21-bus system depicted in Figure 4 detail the following: (i) the load variations directly impact the total number of iterations to reach the desired convergence. For example, when the load is 60% of the nominal value, 10 iterations are required, whereas in the benchmark case, 13 iterations are used, and when the load is 1.8 times the nominal value, 26 iterations are employed to solve the power flow problem. (ii) For all the load increments, it is observed that the convergence rate of the triangular-based bipolar power flow is linear, implying that under normal operating conditions, this method can ensure convergence to the power flow solution. However, this is only possible if the system is operated far from the voltage collapse point.

### 5.2. Results in the 85-Bus Grid

After the implementation of the proposed triangular-based method for bipolar DC grids in the 85-bus system under the nominal operating conditions, we found that (i) when the neutral wire is solidly grounded in all the nodes of the grid, then the total power losses is 452.2981 kW, i.e., 6.76% of the total power consumption, while when the neutral wire is non-grounded, the total power increases to 489.5759 kW, i.e., 7.32% of the net power consumption; (ii) the total number of iterations was about 10 for the solidly-grounded case and 13 for the non-grounded case.

**Figure 5:**
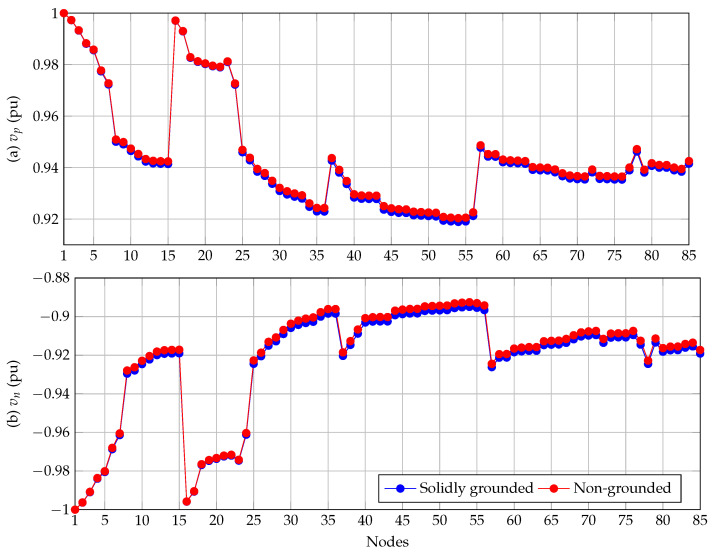
Voltage behavior for the 85-bus grid considering solidly-grounded and non-grounded neutral wire.

On the other hand, in Figure 5 are presented the voltage profiles in the 85-bus system for the positive and negative poles considering solidly-grounded and non-grounded operation cases.

Voltage profiles in Figure 5 show that (i) for the solidly-grounded operative costs, the minimum voltage in the positive pole is 0.9189 pu at node 54, and for the negative pole it is 0.8950 pu at the same node; and (ii) in the non-grounded scenario, these values are 0.9204 pu for the positive pole, and 0.8925 pu for the negative pole.

Note that when positive and negative voltage profiles are compared (see Figure 5), it is observed that the positive pole has similar behavior to the negative pole, which is very similar to the reflection of the voltage profile with respect to the axis of nodes; even if the system is perfectly balanced, $v_{p}$ must be equal to the absolute value of $v_{n}$.

### 5.3. Complementary Analysis

The following results were also obtained for both test feeders:

✔ In the solidly grounded scenario after 1000 consecutive evaluations of the power flow methodology, the average processing times for the 21- and 85-bus grids were 0.6498 ms and 6.4858 ms, respectively. In the non-grounded case, these times were 0.7318 and 6.7226 ms. Note that the increments in these times are expected since the number of iterations increases in the non-grounded neutral wire simulation scenario.
✔ Comparative simulations with the classical backward/forward power flow method adapted for bipolar DC grids demonstrated that both methodologies reach the same voltage profiles and power losses calculations. However, the main advantage of the triangular-based power flow method is the processing time required, since for both tests feeders were 20 % faster than the backward/forward power flow method, which confirmed the results presented by authors of [15] in the case of monopolar DC grid equivalents.

## 6. Conclusions and Future Works

The power flow problem in bipolar DC grids with multiple constant power loads was addressed in this research through the application of the triangular-based power flow formulation. The proposed formulation is applicable in radial configurations, considering the possibility that the neutral cable is or is not grounded in all the nodes of the system. The numerical results in the 21-bus system demonstrate that when the neutral pole is solidly grounded, the maximum voltage regulation bound under normal operative conditions was about 10.99% and total grid power losses about 91.2701 kW; conversely, for the non-grounded simulation case, the voltage regulation of the network was 11.17% and total grid losses increased to 95.4237 kW. Numerical evaluations where the total load was incremented from 60% to 180% of the nominal value illustrated that the convergence of the triangular-based power flow method is linear, and the effect of the load increments is transferred to the number of iterations.

In the case of the 85-bus grid, the minimum voltage profile occurs in the negative pole for the solidly-grounded and non-grounded operative scenarios with magnitudes of 0.8950 pu and 0.8925 pu, respectively; while in the case of the power losses, the solidly-grounded case has a total power loss of 452.2981 kW, which increased to 489.5759 kW in the non-grounded scenario.

For future research, it will be possible to execute the following works: (i) propose a recursive power solver for bipolar DC grids by linearizing the demanded currents around the initial voltage values and then updating this point until the desired convergence error is reached; (ii) determine via metaheuristic or convex optimization the optimal subset of nodes where the neutral cable must be solidly grounded to reduce the total grid power losses; and (iii) design an experimental scenario where the power flow methodologies for DC bipolar networks can be validated.

## Figures and Tables

**Table 1:** Data for the 21-bus system (all powers in kW).

Node *j*	Node *k*	$R_{jk}$ (Ω)	$P_{dk,po}$	$P_{dk,no}$	$P_{dk,pn}$
1	2	0.053	70	100	0
1	3	0.054	0	0	0
3	4	0.054	36	40	120
4	5	0.063	4	0	0
4	6	0.051	36	0	0
3	7	0.037	0	0	0
7	8	0.079	32	50	0
7	9	0.072	80	0	100
3	10	0.053	0	10	0
10	11	0.038	45	30	0
11	12	0.079	68	70	0
11	13	0.078	10	0	75
10	14	0.083	0	0	0
14	15	0.065	22	30	0
15	16	0.064	23	10	0
16	17	0.074	43	0	60
16	18	0.081	34	60	0
14	19	0.078	9	15	0
19	20	0.084	21	10	50
19	21	0.082	21	20	0

**Table 2:** Data for the 85-bus system (all powers in kW).

Node *j*	Node *k*	$R_{jk}$ (Ω)	$P_{dk,po}$	$P_{dk,no}$	$P_{dk,pn}$	Node *j*	Node *k*	$R_{jk}$ (Ω)	$P_{dk,po}$	$P_{dk,no}$	$P_{dk,pn}$
1	2	0.108	0	0	10.075	34	44	1.002	17.64	17.995	0
2	3	0.163	50	0	40.35	44	45	0.911	50	17.64	17.995
3	4	0.217	28	28.565	0	45	46	0.911	25	17.64	17.995
4	5	0.108	100	50	0	46	47	0.546	7	7.14	10
5	6	0.435	17.64	17.995	25.18	35	48	0.637	0	10	0
6	7	0.272	0	8.625	0	48	49	0.182	0	0	25
7	8	1.197	17.64	17.995	30.29	49	50	0.364	18.14	0	18.505
8	9	0.108	17.8	350	40.46	50	51	0.455	28	28.565	0
9	10	0.598	0	100	0	48	52	1.366	30	0	15
10	11	0.544	28	28.565	0	52	53	0.455	17.64	35	17.995
11	12	0.544	0	40	45	53	54	0.546	28	30	28.565
12	13	0.598	45	40	22.5	52	55	0.546	38	0	48.565
13	14	0.272	17.64	17.995	35.13	49	56	0.546	7	40	32.14
14	15	0.326	17.64	17.995	20.175	9	57	0.273	48	35.065	10
2	16	0.728	17.64	67.5	33.49	57	58	0.819	0	50	0
3	17	0.455	56.1	57.15	50.25	58	59	0.182	18	28.565	25
5	18	0.820	28	28.565	200	58	60	0.546	28	43.565	0
18	19	0.637	28	28.565	10	60	61	0.728	18	28.565	30
19	20	0.455	17.64	17.995	150	61	62	1.002	12.5	29.065	0
20	21	0.819	17.64	70	152.5	60	63	0.182	7	7.14	5
21	22	1.548	17.64	17.995	30	63	64	0.728	0	0	50
19	23	0.182	28	75	28.565	64	65	0.182	12.5	25	37.5
7	24	0.910	0	17.64	17.995	65	66	0.182	40	48.565	33
8	25	0.455	17.64	17.995	50	64	67	0.455	0	0	0
25	26	0.364	0	28	28.565	67	68	0.910	0	0	0
26	27	0.546	110	75	175	68	69	1.092	13	18.565	25
27	28	0.273	28	125	28.565	69	70	0.455	0	20	0
28	29	0.546	0	50	75	70	71	0.546	17.64	38.275	17.995
29	30	0.546	17.64	0	17.995	67	72	0.182	28	13.565	0
30	31	0.273	17.64	17.995	0	68	73	1.184	30	0	0
31	32	0.182	0	175	0	73	74	0.273	28	50	28.565
32	33	0.182	7	7.14	12.5	73	75	1.002	17.64	6.23	17.995
33	34	0.819	0	0	0	70	76	0.546	38	48.565	0
34	35	0.637	0	0	50	65	77	0.091	7	17.14	25
35	36	0.182	17.64	0	17.995	10	78	0.637	28	6	28.565
26	37	0.364	28	30	28.565	67	79	0.546	17.64	42.995	0
27	38	1.002	28	28.565	25	12	80	0.728	28	28.565	30
29	39	0.546	0	28	28.565	80	81	0.364	45	0	75
32	40	0.455	17.64	0	17.995	81	82	0.091	28	53.75	0
40	41	1.002	10	0	0	81	83	1.092	12.64	32.995	62.5
41	42	0.273	17.64	25	17.995	83	84	1.002	62	72.2	0
41	43	0.455	17.64	17.995	0	13	85	0.819	10	10	10

**Table 3:** Voltage for both simulation cases in the 21-bus system.

Node	+Pole (V)	0 Pole (V)	−Pole (V)
Grounded neutral			
1	1000	0	−1000
2	996.2761	0	−994.6716
3	960.0683	0	−968.4100
4	952.3714	0	−962.7830
5	952.1067	0	−962.7830
6	950.4396	0	−962.7830
7	953.7448	0	−964.5412
8	951.0867	0	−960.4284
9	943.8619	0	−960.7609
10	937.4888	0	−948.4698
11	930.9220	0	−942.8952
12	925.1152	0	−936.9933
13	926.9467	0	−939.7613
14	916.4715	0	−930.2940
15	905.4444	0	−921.0085
16	896.1420	0	−913.9505
17	890.1027	0	−911.4861
18	893.0582	0	−908.6017
19	909.9528	0	−924.3558
20	905.7061	0	−921.1448
21	908.0565	0	−922.5781
Non-grounded neutral			
1	1000	0	−1000
2	996.2821	−1.6193	−994.6628
3	959.5205	9.2157	−968.7363
4	951.7636	11.3722	−963.1358
5	951.4955	11.6403	−963.1358
6	949.8030	13.3327	−963.1358
7	953.1183	11.7700	−964.8884
8	950.4291	10.3922	−960.8213
9	943.1110	17.9963	−961.1073
10	936.5746	12.4855	−949.0602
11	929.9259	13.6204	−943.5464
12	924.0247	13.7095	−937.7342
13	925.9357	14.4762	−940.4119
14	915.1628	15.9904	−931.1532
15	903.9040	18.1140	−922.0181
16	894.4081	20.6576	−915.0657
17	888.2594	24.3408	−912.6002
18	891.2522	18.5786	−909.8309
19	908.5513	16.7359	−925.2872
20	904.2616	17.8323	−922.0939
21	906.6158	16.9276	−923.5434

## Data Availability

No new data were created or analyzed in this study. Data sharing is not applicable to this article.
